# The Complex Bidirectional Relationship Between Aging and Atherosclerosis: Mechanistic Insights and Translational Opportunities

**DOI:** 10.14336/AD.2025.10620

**Published:** 2026-06-20

**Authors:** Binquan You, Dong Yi, Bingyin Wang, Hua Yan

**Affiliations:** ^1^Departments of Cardiology, Suzhou Kowloon Hospital, School of Medicine Shanghai Jiaotong University, Suzhou, China; ^2^Department of Cardiology, Wuhan Asia Heart Hospital, Wuhan University of Science and Technology, Wuhan, China

**Keywords:** Aging, Atherosclerosis, Endothelial Dysfunction, Immune Senescence; Mitochondrial Oxidative Stress, Senescence-Associated Secretory Phenotype (SASP)

## Abstract

Aging is a major independent risk factor for atherosclerosis (AS), which in turn can accelerate systemic aging, thereby creating a self-reinforcing pathological cycle. These two processes share fundamental molecular mechanisms, such as endothelial dysfunction, phenotypic switching of vascular smooth muscle cells (VSMCs), immune senescence, mitochondrial oxidative stress, and the senescence-associated secretory phenotype (SASP). These overlapping pathways promote chronic inflammation and vascular injury, thereby advancing AS progression and contributing to systemic physiological decline. This review examines the shared mechanisms underlying aging and AS and evaluates emerging therapeutic strategies aimed at disrupting this bidirectional relationship, including senolytics, anti-inflammatory agents, and NAD⁺ supplementation. Targeting these converging pathways presents a promising strategy for mitigating cardiovascular disease and extending the health span.

## 1. Introduction

**Figure 1.**
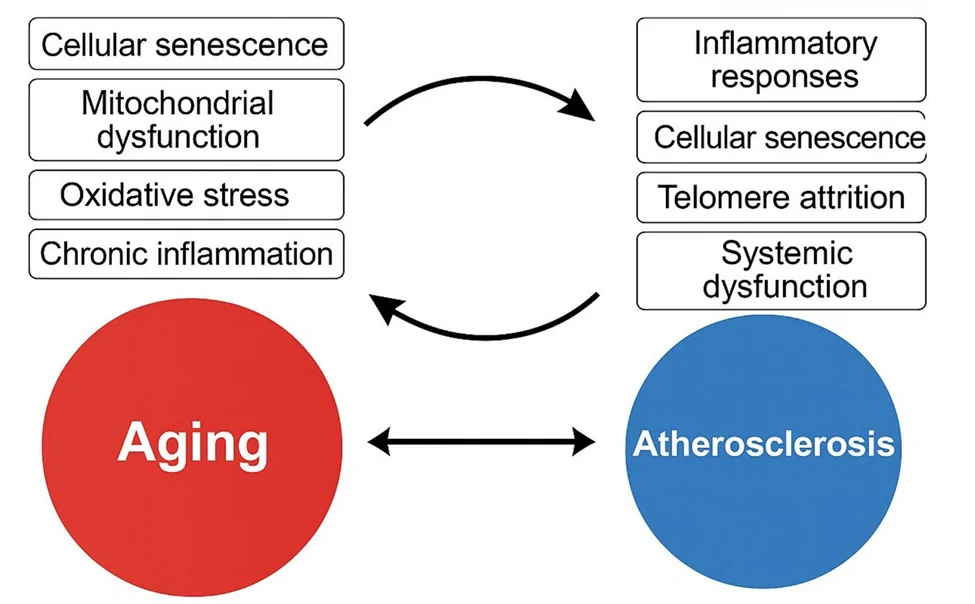
**Biological Interactions Between Aging and Atherosclerosis.** This schematic illustrates the bidirectional interplay between aging and atherosclerosis (AS) through shared cellular and molecular mechanisms. On the left, biological aging promotes vascular dysfunction via endothelial cell (EC) senescence, mitochondrial dysfunction, chronic inflammation, and senescence-associated secretory phenotype (SASP), all contributing to a pro-atherogenic microenvironment. On the right, progression of AS exacerbates systemic aging by inducing tissue ischemia, telomere attrition, immune dysregulation, and stem cell exhaustion. Central feedback loops highlight how these two processes reinforce one another.

Population aging is a defining demographic trend of the 21st century, with profound implications for public health and healthcare systems worldwide. As life expectancy increases, the burden of chronic, age-related diseases—most notably cardiovascular diseases—are rising at an unprecedented rate [1]. Among these, atherosclerosis (AS) remains a leading cause of morbidity and mortality globally. The relationship between aging and AS is not merely associative but deeply mechanistic, involving a complex interplay of biological processes that drive both vascular degeneration and systemic decline [2, 3]. Traditionally, AS was considered a degenerative condition resulting from lipid accumulation and mechanical injury to the arterial wall [4]. However, recent advances in molecular biology and systems medicine have redefined AS as a multifactorial disease rooted in chronic inflammation, immune dysfunction, oxidative stress, and cellular senescence—all hallmarks of aging [5, 6]. These shared features suggest that aging and AS are not independent phenomena but intimately linked through overlapping molecular pathways (Fig. 1) [7, 8].

Aging affects every component of the vascular system. Endothelial cells exhibit impaired nitric oxide production and increased adhesion molecule expression; vascular smooth muscle cells (VSMCs) undergo phenotypic switching; immune cells become dysregulated; and mitochondria accumulate oxidative damage [9-12]. These age-related alterations create a pro-atherogenic environment that promotes plaque formation and instability. Conversely, the chronic progression of AS accelerates biological aging by exacerbating inflammation, promoting tissue hypoxia, and impairing stem cell and progenitor cell function [13, 14]. Understanding this bidirectional relationship is crucial for identifying shared therapeutic targets. This review examines the cellular and molecular mechanisms linking aging and AS, evaluates emerging therapeutic interventions, and discusses the translational implications of targeting aging pathways to combat cardiovascular disease.

## 2. Mechanisms by Which Aging Promotes AS

The aging vasculature undergoes a constellation of structural, functional, and molecular changes that converge to promote AS. With chronological aging, the risk of developing AS increases exponentially, independent of classical risk factors such as hypertension, dyslipidemia, or diabetes [15]. This relationship is mediated by cellular senescence, mitochondrial dysfunction, impaired repair capacity, and dysregulated inflammatory signaling—core hallmarks of vascular aging that synergistically accelerate atherogenesis. Emerging research reveals that these pathways form interconnected networks, creating a self-amplifying cycle of vascular deterioration.

### 2.1 Endothelial Dysfunction: The Molecular Gateway to AS

One of the earliest hallmarks of vascular aging is endothelial dysfunction, marked by reduced bio-availability of nitric oxide (NO), a vasoprotective molecule critical for maintaining vascular tone, anti-thrombotic properties, and anti-inflammatory homeostasis [16]. Age-related increases in reactive oxygen species (ROS)—generated from dysfunctional mitochondria and NADPH oxidase activation—rapidly degrade NO via peroxynitrite formation, diminishing its vasodilatory effects [17]. This is accompanied by the activation of redox-sensitive transcription factors such as NF-κB, which upregulate adhesion molecules (ICAM-1, VCAM-1) and chemokines (MCP-1), thereby promoting monocyte adhesion and transendothelial migration [18]. These processes constitute the initial steps in fatty streak formation.

Notably, aging alters NO synthase (NOS) inform dynamics: Constitutive NOS (cNOS) activity increases in aged vasculature due to post-translational modifications (e.g., phosphorylation), while endothelial NOS (eNOS) uncoupling further reduces NO output. This paradox—elevated enzymatic activity yet reduced functional NO—highlights the complexity of age-related endothelial dysregulation [19, 20]. Additionally, endothelial-to-mesenchymal transition (EndMT), amplified in aging, contributes to fibrotic remodeling and loss of endothelial integrity via TGF-β/Smad signaling, promoting plaque development and instability through α-SMA-positive fibroblast-like cell accumulation [21].

Aging significantly impacts VSMCs, shifting them from a quiescent, contractile phenotype to a synthetic, pro-inflammatory state. This phenotypic switch is marked by increased secretion of extracellular matrix (ECM) proteins, collagen, fibronectin, and matrix metallo-proteinases (MMPs), contributing to arterial stiffening, intimal thickening, and plaque vulnerability [22].

### 2.2 Mitochondrial Dysfunction and Oxidative Damage

Mitochondria in aging vascular cells undergo structural degeneration (swelling, cristae loss) and functional decline, leading to impaired oxidative phosphorylation, diminished ATP production, and increased mitochondrial ROS (mtROS) [23, 24]. Elevated mtROS further damages mitochondrial DNA (mtDNA), creating a vicious cycle. Leaked mtDNA acts as a danger-associated molecular pattern (DAMP), activating innate immune sensors (TLR9, cGAS-STING) and triggering NLRP3 inflammasome assembly, which amplifies IL-1β/IL-18-driven inflammation [25]. Aging also depletes NAD⁺ pools, compromising sirtuin activity (SIRT1, SIRT3). SIRT1 deacetylates NF-κB and PGC-1α, regulating inflammation and mitochondrial biogenesis, while SIRT3 maintains antioxidant defenses (SOD2). Their decline accelerates redox imbalance. Critically, mitophagy impairment (e.g., reduced PINK1/Parkin signaling) allows accumulation of damaged mitochondria, a feature exacerbated by IL-6-mediated suppression of autophagy pathways [26]. This nexus between mitochondrial failure and inflammation is now recognized as a hallmark of vascular aging.

Telomere shortening is a well-recognized hallmark of biological aging. Each cell division shortens telomeres unless maintained by telomerase, which is silenced in somatic vascular cells. In endothelial cells and VSMCs, critically short telomeres trigger replicative senescence *via* p53/p21 activation, halting proliferation and promoting a pro-inflammatory, pro-thrombotic phenotype. Telomere attrition is not merely a biomarker but a functional contributor: Telomerase-deficient mice exhibit accelerated plaque formation, impaired endothelial regeneration, and increased oxidative stress. Clinical data reveal shorter leukocyte telomeres in AS patients versus age-matched controls, and telomere length inversely correlates with plaque burden. Epigenetic modifications (e.g., histone methylation at telomeric regions) further accelerate attrition [1]. This process creates a senescent cell burden that exacerbates vascular remodeling and limits repair capacity.

### 2.3 Immunosenescence and gene Regulation

Aging profoundly reshapes the immune system, a phenomenon termed immunosenescence [27, 28]. This is characterized by a decline in adaptive immunity and the emergence of a chronic low-grade inflammatory state known as inflammaging. In inflammaging, terminally differentiated effector memory T cells expand, thymic involution reduces naïve T cell production, and circulating pro-inflammatory cytokines such as IL-6, TNF-α, and CRP increase [29]. Innate immunity is also dysregulated in aging. Aged macrophages and dendritic cells exhibit altered polarization, impaired phagocytic activity, and enhanced inflammasome activation (e.g., NLRP3). These changes promote foam cell formation and lesion expansion [30]. For instance, aged macrophages are more prone to engulf oxidized low-density lipoprotein (ox-LDL), transforming into foam cells. The accumulation of foam cells within plaques enlarges the lipid core, destabilizing the plaque [31].

Specific methylation patterns in aging endothelial cells and VSMCs alter the transcription of genes involved in inflammation, lipid metabolism, and apoptosis [32]. For example, hypermethylation of certain gene promoters may suppress the expression of anti-inflammatory and antioxidant genes, while hypomethylation may activate pro-inflammatory gene expression. Age-related disruption of histone acetylation homeostasis alters chromatin accessibility and contributes to abnormal expression of cytokines and adhesion molecules. MicroRNAs (e.g., miR-34a, miR-146a) and long non-coding RNAs (e.g., MALAT1) are dysregulated in vascular aging and play critical roles in endothelial inflammation, VSMC proliferation, and vascular remodeling. These epigenetic changes do not merely reflect aging but actively shape cellular phenotypes and susceptibility to AS [33].

### 2.4 Stem Cell Exhaustion and Repair Deficit

Aging depletes the vascular progenitor pool, including endothelial progenitor cells (EPCs) and mesenchymal stem cells (MSCs), impairing endothelial repair and angiogenesis. EPCs from aged individuals exhibit reduced migratory capacity, senescent morphology, and decreased nitric oxide synthase expression [34]. Similarly, aged MSCs show impaired paracrine signaling, increased senescence markers (e.g., p16^INK4a, p21), and diminished capacity for vascular regeneration [35]. This repair deficit, combined with chronic exposure to inflammatory and pro-oxidant stimuli, limits the vessel's ability to recover from injury and adapt to hemodynamic stress—key features of advanced AS [36]. In young individuals, vascular progenitor cells can quickly migrate to damaged vascular sites to repair endothelial injuries and restore vascular function. However, in aged individuals, the number and function of these cells decline, prolonging the repair process and allowing more lipids and inflammatory cells to accumulate at the injury site, eventually leading to plaque formation and progression.

Recent studies implicate altered calcium handling in vascular aging: Dysregulated Ca^2^⁺/calmodulin-dependent pathways in VSMCs enhance contractility and promote calcification [37]. Additionally, clonal hematopoiesis of indeterminate potential (CHIP), age-related expansion of mutated hematopoietic stem cells (e.g., TET2, DNMT3A), exacerbates AS through IL-1β/IL-6 over-production. These pathways represent promising therapeutic targets for "geroscience"-guided AS interventions [38].

## 3. How AS Accelerates Biological Aging

While chronological aging is widely recognized as a primary risk factor for AS, emerging evidence increasingly supports a bidirectional relationship: AS itself functions not only as a disease of aging but also as a potent accelerant of biological aging. This reciprocal interaction is particularly evident when examining the systemic consequences of prolonged arterial dysfunction. As atherosclerotic plaques progressively narrow arterial lumens and destabilize vascular integrity, the ensuing chronic ischemia deprives downstream tissues, particularly those with high metabolic demands such as the brain, heart, kidneys, and skeletal muscles, of sufficient oxygen and nutrients [6]. This sustained hypoperfusion initiates and perpetuates functional declines that are phenotypically indistinguishable from those attributed to natural aging, including cognitive impairment, renal insufficiency, sarcopenia, and frailty [39].

### 3.1 Vasculature-Tissue Axis in Ischemic Aging: Chronic Hypoperfusion Drives Multi-Organ Dysfunction

The progression of atherosclerotic plaques leads to progressive vascular stenosis and flow limitation, causing persistent chronic ischemia in downstream tissues. This hypoperfusive state has particularly severe consequences for organs with high metabolic demands, such as the brain, heart, kidneys, and skeletal muscle. Histological studies indicate that chronic hypoxia induces mitochondrial dysfunction, manifested by reduced activity of respiratory chain complexes (especially Complex IV) and diminished ATP synthesis. This forces cells to shift towards glycolysis for energy production, further increasing oxidative stress burden [40]. This metabolic shift is particularly pronounced in neural tissue, leading to an energy crisis in hippocampal neurons and synaptic dysfunction, thereby accelerating cognitive decline and neurodegeneration [41]. Renal microcirculatory impairment presents as progressive decline in glomerular filtration rate (GFR) and tubulointerstitial fibrosis. Renal tissue from clinical AS patients exhibits features of premature aging, including increased glomerulosclerosis, accelerated podocyte loss, and mitochondrial vacuolization in tubular epithelial cells. In the muscular system, chronic ischemia mediates skeletal muscle atrophy through inhibition of the mTORC1 signaling pathway and upregulation of myostatin, clinically manifesting as characteristic reduced muscle strength and diminished exercise tolerance [42].

Notably, this ischemia-induced tissue dysfunction is not limited to areas of macrovascular disease. Recent research has revealed that microcirculatory disturbances play a pivotal role in AS-associated aging. Damage to the endothelial glycocalyx and capillary rarefaction increase the oxygen diffusion distance within tissues. This creates a microenvironment of "physiological hypoxia" even in vascular areas without complete occlusion, promoting fibrosis and parenchymal cell apoptosis through sustained activation of the HIF-1α pathway [43].

### 3.2 Inflammation-Immune System Cascade: From Local Plaque to Systemic Inflammaging

A fundamental hallmark of AS is its chronic inflammatory milieu. Even in its earliest stages, AS is marked by endothelial dysfunction, which promotes the adhesion and transmigration of immune cells such as monocytes and T lymphocytes into the intima. These immune cells differentiate into foam cells and perpetuate the inflammatory cascade through the release of cytokines (e.g., TNF-α, IL-1β) and chemokines (e.g., MCP-1), along with reactive oxygen species (ROS) and damage-associated molecular patterns (DAMPs) [44]. These signaling molecules do not remain confined to the vascular wall but disseminate throughout the circulatory system, exerting pro-senescent effects in distal tissues. This systemic inflammatory signaling is mechanistically linked to multiple age-related pathologies, including neurodegeneration, insulin resistance, musculoskeletal decline, and immune senescence [45].

One of the most deleterious consequences of AS-induced inflammation is the acceleration of cellular senescence. Senescent cells enter a state of irreversible growth arrest accompanied by the secretion of a pro-inflammatory and matrix-degrading profile known as the senescence-associated secretory phenotype (SASP) [5]. The presence of senescent cells in various organs contributes to tissue dysfunction, impaired regeneration, and chronic inflammation, thereby forming a vicious cycle that exacerbates aging [46]. In the context of AS, endothelial cells, vascular smooth muscle cells, and infiltrating immune cells all exhibit features of premature senescence, driven by sustained exposure to oxidative stress, DNA damage, and telomere attrition [11].

### 3.3 Stem Cell Exhaustion and Regenerative Failure: Systemic Collapse of Repair Mechanisms

At the level of stem cell biology, AS exerts a profound impact on the functionality and longevity of key progenitor cell populations. Hematopoietic stem cells (HSCs), responsible for the lifelong replenishment of blood and immune cells, display altered behavior when exposed to an atherogenic environment. This includes a skewing toward the myeloid lineage at the expense of lymphoid cells, a bias that both contributes to and reflects systemic inflammaging [47]. Additionally, chronic inflammation impairs HSC self-renewal, leading to clonal hematopoiesis of CHIP, a condition associated with an increased risk of cardiovascular events and all-cause mortality [48]. MSCs, which play critical roles in tissue repair, angiogenesis, and immunomodulation, are likewise impaired in AS. These cells exhibit reduced proliferation, increased oxidative DNA damage, and a diminished ability to differentiate into endothelial and smooth muscle cells. Consequently, the capacity to repair vascular injury and regenerate damaged tissues is significantly compromised, contributing to cumulative organ dysfunction and physical decline commonly observed in elderly populations with cardiovascular disease [49].

AS also contributes to biological aging through its influence on the epigenome. Persistent inflammation and metabolic dysregulation induce aberrant DNA methylation patterns, histone modifications, and non-coding RNA expression, leading to dysregulation of genes involved in stress responses, cellular proliferation, and longevity [50, 51]. For example, hypermethylation of anti-inflammatory and antioxidant genes can perpetuate the pro-aging environment, while altered expression of genes like p16^INK4a and p53 can accelerate senescence and impair regenerative responses. These epigenetic changes are not merely downstream effects but active contributors to the aging phenotype observed in individuals with advanced cardiovascular disease [52].

### 3.4 Telomere Shortening and Mitochondrial Dysfunction

Another pivotal mechanism through which AS accelerates biological aging is via telomere shortening. Telomeres, the protective caps at the ends of chromosomes, shorten progressively with each cell division, and this process is hastened in the presence of oxidative stress and chronic inflammation, both of which are abundant in atherosclerotic conditions [12]. In endothelial cells and leukocytes, telomere shortening is not merely a biomarker of aging but a driver of replicative senescence, impaired vascular repair, and increased apoptosis [53]. This cellular attrition contributes directly to tissue aging and loss of homeostasis, correlating with greater biological age and reduced life expectancy [54].

Concomitant with telomere dysfunction, mitochondrial impairment plays a central role in aging, exacerbated by AS [55]. Mitochondria are particularly susceptible to oxidative damage due to their role in energy production and ROS generation. In atherosclerotic tissues, damaged mitochondria produce excessive ROS while simultaneously suffering declines in ATP output, mitophagy efficiency, and NAD⁺ availability [56]. Reduced NAD⁺ levels impair the activity of sirtuins—a family of NAD⁺-dependent enzymes involved in DNA repair, chromatin remodeling, and cellular stress resistance, thus compromising cellular longevity mechanisms and exacerbating age-associated cellular dysfunction [57].

In synthesis, AS is not simply an age-related pathology but an active driver of systemic aging. Through a complex interplay of chronic inflammation, oxidative stress, cellular senescence, telomere attrition, stem cell dysfunction, mitochondrial compromise, and epigenetic reprogramming, AS accelerates the biological aging process across multiple organ systems. Recognizing this multidimensional impact emphasizes the importance of early preventive strategies and therapeutic interventions targeting vascular health to mitigate cardiovascular risk, preserve systemic vitality, and extend health span in aging populations.

## 4. Interventional Strategies and Translational Advances

**Figure 2.**
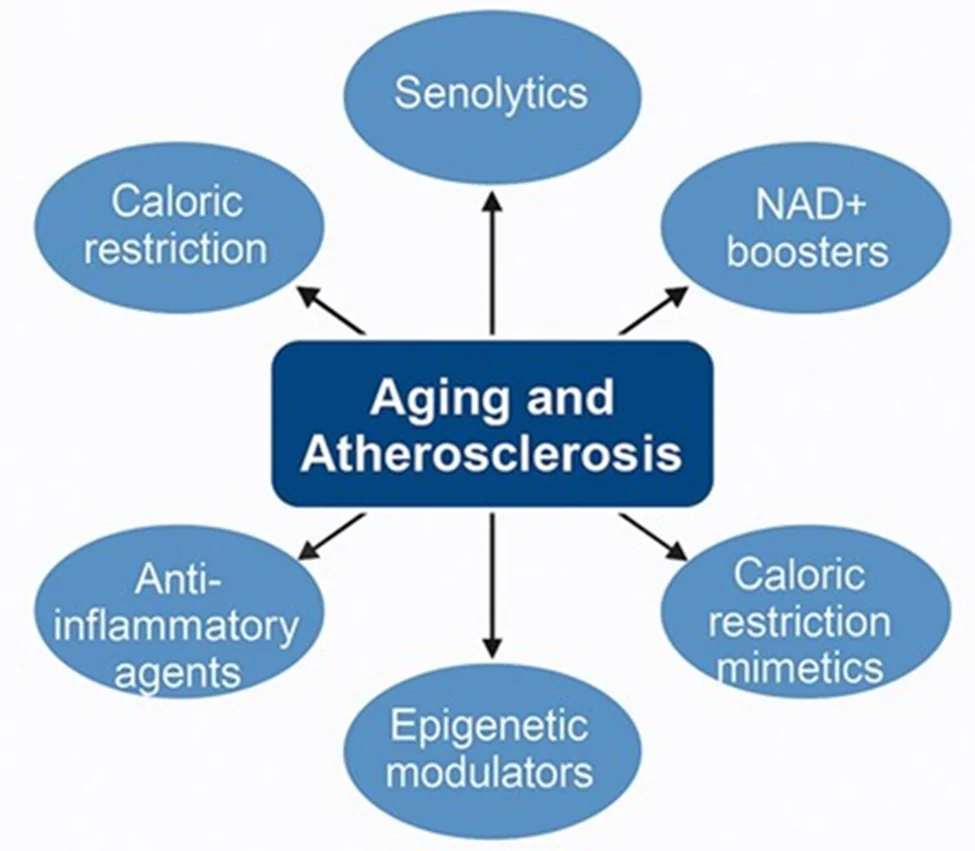
**Summary of Intervention Strategies.** This diagram presents a flowchart-style summary of intervention strategies including senolytics, NAD⁺ boosters, anti-inflammatory agents, and epigenetic modulators, all directed at disrupting the feedback loop between aging and vascular disease. Senolytics (e.g., Dasatinib + Quercetin, Fisetin) are agents that selectively eliminate senescent cells to reduce inflammation and improve vascular function. NAD⁺ boosters (such as NMN and NR) enhance mitochondrial activity and sirtuin signaling, promoting cellular repair and metabolic resilience. Anti-inflammatory drugs (e.g., Canakinumab, colchicine) target cytokine pathways like IL-1β and IL-6 to mitigate plaque progression. Epigenetic modulators, including HDAC and BET inhibitors, aim to restore youthful gene expression and reduce vascular aging. These interventions may be used independently or in combination to interrupt the aging-atherosclerosis cycle.

The recognition that aging and AS share fundamental molecular hallmarks—including chronic inflammation, mitochondrial dysfunction, impaired autophagy, and genomic instability has catalyzed a paradigm shift in cardiovascular medicine [58]. This convergence of geroscience and cardiology has revealed that targeting the biological underpinnings of aging may simultaneously disrupt AS pathogenesis. Consequently, therapeutic goals have evolved beyond mere cardiovascular risk reduction toward extending health span and delaying systemic age-associated decline through interventions with dual cardioprotective and geroprotective effects (Fig. 2).

### 4.1 Senolytic and Senomorphic Therapies

Senescent cells, driven by their pro-inflammatory SASP, accelerate vascular aging by promoting endothelial dysfunction, calcification, and plaque vulnerability. Senolytics (e.g., dasatinib + quercetin [D+Q]) selectively eliminate these cells [59], while senomorphic modulate SASP without inducing apoptosis [59]. Preclinical studies demonstrate that D+Q reduces vascular senescence markers [59, 60], restores nitric oxide bioavailability, and improves arterial compliance in aged mice [60].The flavonoid fisetin exhibits potent senotherapeutic activity, extending lifespan, suppressing IL-6/TNF-α-driven inflammation, and enhancing collagen stabilization in AS-prone mice [61]. Early-phase human trials (e.g., in idiopathic pulmonary fibrosis) confirm senolytic safety and bioactivity, providing critical translational momentum for cardiovascular applications [62]. However, complexities persist in specific senescent VSMCs that may paradoxically stabilize fibrous caps through extracellular matrix deposition. This highlights the need for tissue-specific senolytic delivery systems to avoid compromising plaque integrity, a key consideration for clinical translation [63].

### 4.2 NAD⁺ Repletion and Mitochondrial Enhancement

Age-related NAD⁺ depletion impairs sirtuin activity (SIRT1/3/6), compromising mitochondrial quality control, DNA repair, and metabolic flexibility. Oral supplementation with NAD⁺ precursors (nicotinamide mononucleotide; NMN) [64] and nicotinamide riboside; NR) boosts intracellular NAD⁺ pools by >50% in preclinical models, reactivating sirtuins to enhance mitochondrial biogenesis, reduce oxidative damage, and improve bioenergetics [65]. In hyperlipidemic mice, NAD⁺ repletion reduces endothelial activation, inhibits NLRP3 inflammasome [66] priming, and increases plaque collagen content [67]. Ongoing trials (NAD+BOOST, MIB-626) [68] quantify NAD⁺ precursors’ effects on vascular stiffness, endothelial function, and metabolic health in elderly cohorts. Combining NAD⁺ boosters with exercise regimens may yield synergistic benefits by amplifying PGC-1α-mediated mitochondrial adaptations, a strategy poised for AS clinical trials [69, 70].

### 4.3 Targeted Anti-inflammatory Therapies

The inflammatory hypothesis of AS has gained robust clinical support through trials targeting specific cytokines. The CANTOS trial was a landmark study demonstrating that canakinumab, an IL-1β monoclonal antibody, significantly reduced recurrent myocardial infarction and cardiovascular mortality in patients with elevated hs-CRP, independent of lipid lowering [71]. Complementary findings from COLCOT and LoDoCo2 trials have established colchicine, a microtubule inhibitor with anti-neutrophilic properties, as an effective secondary prevention agent [72]. Colchicine reduces the activation of the NLRP3 inflammasome [66], lowers IL-6 and CRP levels, and prevents destabilization of atherosclerotic plaques [72, 73]. Emerging agents targeting IL-6, MCP-1 [74], and TNF-α are under investigation. Additionally, ziltivekimab, an IL-6 ligand inhibitor, has shown promise in lowering inflammatory biomarkers in chronic kidney disease patients with elevated cardiovascular risk (RESCUE trial) [75].

### 4.4 Epigenetic and RNA-based Therapies

Given the role of epigenetic alterations in both aging and AS, epigenetic reprogramming has emerged as a frontier in translational research. Histone deacetylase (HDAC) inhibitors and bromodomain and extraterminal (BET) protein inhibitors have shown potential in preclinical models to restore youthful gene expression profiles, inhibit inflammatory signaling, and reduce neointimal formation [76].

Furthermore, non-coding RNAs, such as microRNAs (miRNAs) and long non-coding RNAs (lncRNAs) [77], are being investigated as regulators of vascular aging [78]. For instance, miR-146a and miR-34a are known to modulate inflammation and senescence, while lncRNA MALAT1 may influence endothelial homeostasis. Oligonucleotide-based therapies targeting these RNAs represent a novel and precise approach to modulate vascular biology [78].

### 4.5 Metabolic Modulators and Caloric Restriction Mimetics

Metformin [79], traditionally used for type 2 diabetes, has emerged as a caloric restriction mimetic that activates AMP-activated protein kinase (AMPK) [80, 81] and inhibits mTOR signaling. This improves mitochondrial function and attenuates vascular aging [82]. Observational data suggest metformin use is associated with reduced cardiovascular events and mortality, independent of glycemic control. Rapamycin and its analogs (rapalogs) directly inhibit mTORC1 [82, 83] and have been shown to reduce senescence markers, improve lipid metabolism, and stabilize atherosclerotic plaques in preclinical models. Trials such as PEARL and TAME (Targeting Aging with Metformin) are helping establish the translational potential of these agents [79, 84].

### 4.6 Lifestyle and Behavioral Interventions

Despite pharmacologic advances, lifestyle modification remains foundational for disrupting the aging-AS nexus. Caloric restriction (CR) induces SIRT1 and FOXO3 activation, lowering leptin/adiponectin ratios and enhancing autophagy, yielding 40% reductions in aortic inflammation in primates [85, 86]. Exercise training amplifies these benefits [69, 87]: aerobic activity boosts PGC-1α-mediated antioxidant defenses, while resistance exercise increases FGF21-mediated lipid oxidation, collectively improving endothelial function and arterial distensibility [69, 88]. Polyphenol-rich diets (Mediterranean/DASH) exert multi-target effects: Modulate gut microbiota to reduce TMAO production [89-91]. Activate Nrf2 to counter oxidative stress [92]. Increase NO bioavailability via eNOS phosphorylation. These synergize with pharmacologic interventions, highlighting the need for integrated multimodal strategies [93].

## 5. Scholarly Disagreements Regarding the Bidirectional Relationship Between Aging and AS

The complex and interdependent relationship between aging and AS has spurred a vigorous academic discourse across the cardiovascular and aging biology communities. While substantial evidence supports the view that AS and aging are mechanistically entangled, disagreements persist regarding their interactions’ directionality, primacy, and reversibility directionality, primacy, and reversibility. These debates are crucial for prioritizing therapeutic targets and designing effective interventions. Below, we elaborate on the most significant and unresolved controversies.

### 5.1 Is aging an independent driver of AS, or merely a permissive co-factorƒ

One school of thought posits that aging alone, independent of traditional cardiovascular risk factors, can initiate AS. This perspective is supported by evidence showing that biological aging processes such as telomere attrition, mitochondrial dysfunction, and epigenetic drift directly lead to endothelial dysfunction and vascular remodeling [5, 12, 94]. In support of this, age-related vascular changes, including increased intimal thickness, elastin fragmentation, and arterial stiffness, have been observed even in normolipidemic, non-diabetic animal models. Histological studies in aged mice on standard chow diets demonstrate fibrotic plaque-like lesions in the aortic arch [95].

However, other researchers argue that aging is insufficient to drive AS unless accompanied by pro-atherogenic stressors such as dyslipidemia, insulin resistance, or hypertension. According to this "two-hit" model, aging creates a vulnerable substrate—altered vascular architecture and heightened inflammatory tone—but requires metabolic insults to precipitate full-blown atherogenesis. For example, APOE-knockout mice do not develop significant lesions unless exposed to aging-related mitochondrial stress or high-fat diets, suggesting that age is a risk amplifier rather than a primary driver [96].

### 5.2 Is "inflammaging" a cause or consequence of ASƒ

A key mechanistic dispute centers on the role of chronic low-grade inflammation in AS. Proponents of the "inflammaging-as-cause" hypothesis argue that aging leads to the accumulation of senescent cells, which release SASP components such as IL-6, TNF-α, and MMPs, promoting leukocyte recruitment, endothelial activation, and foam cell formation [45]. These processes are proposed to initiate and perpetuate early atherogenesis. *In vitro* studies show that aged endothelial cells upregulate VCAM-1 and ICAM-1, facilitating monocyte adhesion and transmigration [9].

Contrastingly, other investigators suggest that inflammation is primarily a downstream effect of atherosclerotic lesion progression. According to this view, oxidative stress, lipid oxidation, and necrotic core formation in developing plaques provoke immune responses and local cytokine production. Here, inflammation is seen as a byproduct, rather than a trigger, of plaque biology. These researchers point to the spatial correlation between macrophage infiltration and plaque vulnerability, rather than age per se, as the principal determinant of inflammatory activity [97, 98].

### 5.3 Can targeting senescent cells therapeutically reverse ASƒ

Senolytic therapies, particularly combinations like dasatinib plus quercetin, have shown promise in preclinical studies by reducing senescent cell burden, decreasing vascular stiffness, and lowering inflammatory biomarkers in aged animals and even nonhuman primates [59, 60]. These findings support the idea that senescence is a biomarker and a driver of AS progression.

However, caution is warranted. Some vascular senescent cells, such as senescent VSMCs, may play a stabilizing role in maintaining the fibrous cap that prevents plaque rupture. Their elimination could paradoxically destabilize plaques and increase the risk of thrombosis and acute coronary events [63]. Moreover, systemic senolysis risks off-target effects in tissues where senescence may serve beneficial roles, such as tumor suppression or wound healing.

### 5.4 Is telomere shortening a marker or a causal agent of ASƒ

Telomere shortening has been implicated in cellular senescence and vascular aging. Experimental models involving telomerase-deficient mice (TERC^−/−^) show impaired endothelial regeneration, reduced angiogenesis, and increased atherosclerotic lesion formation [99]. These observations support the "telomere-driven" model, where replicative senescence impairs vascular repair and immune surveillance, thereby promoting AS.

Nonetheless, critics argue that telomere attrition primarily reflects cumulative oxidative damage, not necessarily a causal agent. Epidemiological studies have found individuals with long telomeres who still develop severe AS, suggesting that telomere length may be a biomarker of systemic stress rather than a mechanistic determinant [100, 101]. Moreover, interventions that solely lengthen telomeres without addressing inflammation or lipid accumulation have not consistently yielded vascular benefits in clinical studies.

### 5.5 Should NAD⁺ metabolism or mTOR signaling be prioritized as a therapeutic targetƒ

Supporters of NAD⁺ repletion argue that restoring NAD⁺ levels via nicotinamide mononucleotide (NMN) or nicotinamide riboside (NR) reactivates SIRT1, enhances mitochondrial function, improves DNA repair, and suppresses inflammation. Sinclair’s group has shown that NAD⁺ boosters reverse vascular dysfunction in aged mice and improve endothelial responsiveness [102].

In contrast, others point to the mTORC1 pathway as a more central aging driver. Rapamycin and its analogs (rapalogs) inhibit mTORC1, thereby promoting autophagy, reducing protein aggregation, and slowing cellular senescence. Studies in LDLR−/− mice have demonstrated that rapamycin reduces lesion size and VSMC proliferation, suggesting greater efficacy in plaque modulation [103]. The debate hinges on whether metabolic reprogramming (*via* NAD⁺) or senescence modulation (via mTOR) yields broader and more durable vascular benefits.

### 5.6 Divergent Frameworks Among Leading Research Teams

Differences in research emphasis among key investigators illustrate how theoretical frameworks shape the interpretation of data and therapeutic focus:

1. David Sinclair’s group (Harvard Medical School) promotes a metabolic axis model, emphasizing NAD⁺-SIRT1 pathways as foundational to aging and AS. Their approach integrates epigenetic reprogramming, mitochondrial rejuvenation, and circadian rhythm modulation [104].
2. James Kirkland’s team (Mayo Clinic) advocates cellular senescence as the unifying pathology behind multimorbidity in aging. They prioritize senolytics for reversing AS and have pioneered human trials targeting senescent cell clearance [105].
3. Charalambos Antoniades’s group (University of Oxford) focuses on vascular inflammation as the key convergence point between aging and AS. They highlight translational strategies targeting IL-1β, NLRP3, and oxidative stress, favoring anti-inflammatory biologics like canakinumab as the most clinically actionable interventions [106].

These varying models—metabolic, cellular senescence, and inflammatory—illustrate AS-aging research's rich and still-evolving landscape, where overlapping mechanisms invite different but not mutually exclusive strategies.

## 6. Current Challenges and Future Directions in Research

Future research must integrate basic science with clinical evidence to decode the "vascular code" of aging. Coordinated interdisciplinary studies are essential to unravel the complex mechanisms linking vascular biology and aging [12]. A key challenge involves the heterogeneity of vascular beds, for example, differences between coronary arteries and the aorta, which may differentially contribute to aging and AS. Both focused investigations and integrated analyses across vascular territories are needed to understand these interactions fully.

Another translational bottleneck is the limited applicability of animal models [107]. Differences between aged mice and human vascular aging highlight the need for models that more accurately mimic human physiology. Moreover, NAD⁺ depletion and impaired SIRT1 signaling are increasingly recognized as contributors to mitochondrial dysfunction and vascular senescence, suggesting that metabolic regulation plays a central role in disease progression [12].

Advanced techniques such as single-cell sequencing may identify specific senescent subpopulations relevant to AS. Artificial intelligence could further personalize risk prediction, while multi-omics technologies, including genomics, transcriptomics, metabolomics, and epigenomics, may uncover biomarkers of vascular aging [57]. Precision medicine guided by aging stratification is poised to transform cardiovascular care.

Future priorities include:

1. Identifying reliable biomarkers of vascular aging through multi-omics integration.
2. Employing AI to predict aging trajectories and cardiovascular risk.
3. Conducting multi-center RCTs to evaluate gerotherapeutics in aging populations.
4. Developing combinatorial therapies involving senolytics, metabolic regulators, immune modulators, and epigenetic agents.

**Figure 3.**
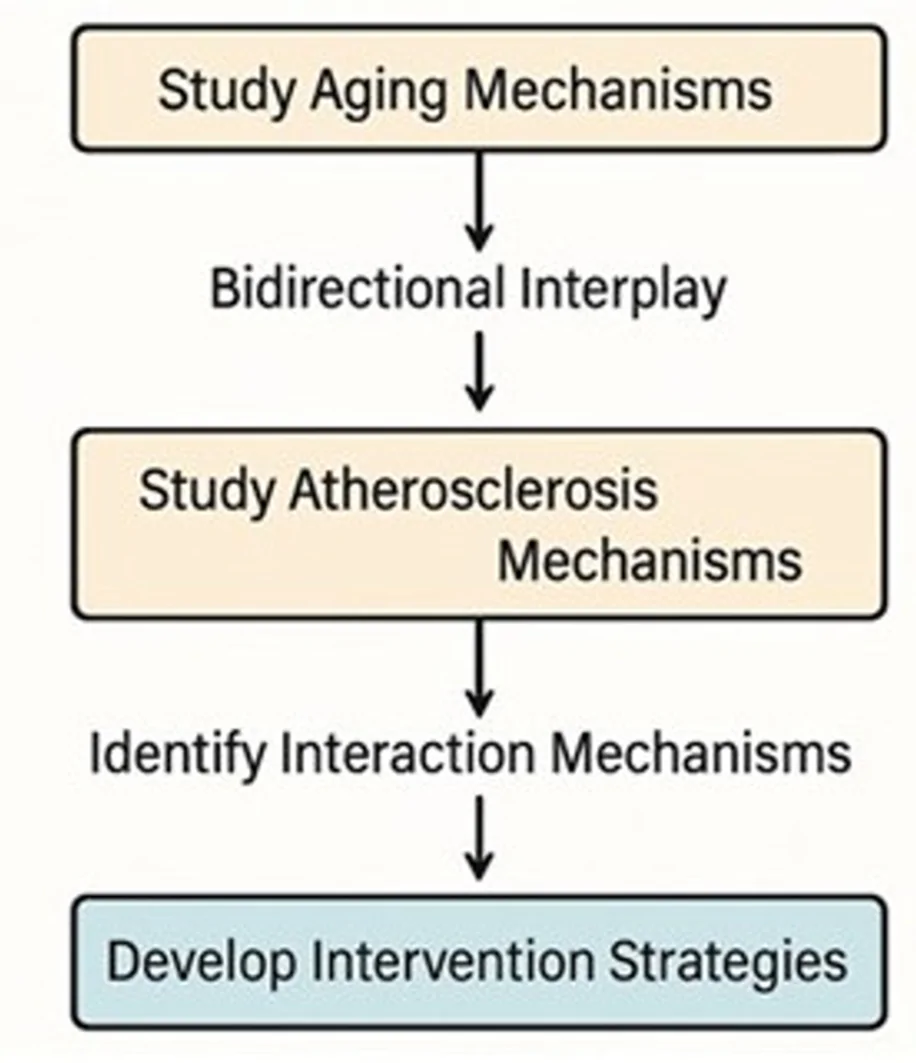
**Research Framework for Understanding the Bidirectional Relationship Between Aging and Atherosclerosis.** This flowchart outlines a stepwise research strategy to explore the mutual reinforcement between aging and atherosclerosis. The process begins with investigating aging mechanisms, including cellular senescence, oxidative stress, and immune remodeling. It proceeds to examine the pathophysiological features of atherosclerosis, such as endothelial dysfunction and vascular inflammation. The central focus is to identify the bidirectional mechanisms linking the two processes. This framework culminates in developing targeted intervention strategies to delay vascular aging and prevent atherosclerotic disease progression.

Ultimately, targeting vascular aging represents a paradigm shift from disease treatment to healthspan extension (Fig 3).

## 7. Conclusions and Perspectives

Aging and AS are intertwined biological processes mutually reinforcing each other through shared molecular mechanisms, including oxidative stress, chronic low-grade inflammation, mitochondrial dysfunction, cellular senescence, SASP, immune remodeling, and epigenetic drift. Disentangling this complex relationship enhances our understanding of vascular pathology and reveals new therapeutic targets. Modern geroscience has shifted the therapeutic focus from symptom management to targeting the root causes of aging. Preclinical studies suggest that senolytics (e.g., Dasatinib + Quercetin, Fisetin), NAD⁺ precursors (e.g., NMN, NR), mTOR inhibitors (e.g., rapamycin), and anti-inflammatory agents (e.g., canakinumab, low-dose colchicine) may delay vascular aging and attenuate AS. Epigenetic modulators and microRNA-targeted therapies are also emerging areas of interest.

Two ongoing debates merit attention. First, whether aging is an independent driver of AS or a permissive co-factor, evidence supports both perspectives: aged models show vascular pathology without external stimuli, but other studies indicate additional insults (e.g., dyslipidemia) are necessary [108, 109]. Second, the causality between inflammation and aging remains under investigation. Some posit that inflammaging initiates AS via cytokine-driven endothelial activation, while others suggest inflammation results from oxidative stress and plaque development [107]. Continued exploration of these controversies and advances in translational medicine offers a promising path toward mitigating vascular aging and extending health span.
